# Strong Coupling between a Single Quantum Emitter and a Plasmonic Nanoantenna on a Metallic Film

**DOI:** 10.3390/nano12091440

**Published:** 2022-04-23

**Authors:** Shun Cao, Yuxin Xing, Yuwei Sun, Zhenchao Liu, Sailing He

**Affiliations:** 1 Centre for Optical and Electromagnetic Research, National Engineering Research Center for Optical Instruments, Zhejiang University, Hangzhou 310058, China; caoshun@zju.edu.cn (S.C.); y.xing@zju.edu.cn (Y.X.); sunyuwei@zju.edu.cn (Y.S.); lzc_archer@zju.edu.cn (Z.L.); 2 Shanghai Institute for Advanced Study, Zhejiang University, Shanghai 200135, China; 3 Department of Electromagnetic Engineering, School of Electrical Engineering, KTH Royal Institute of Technology, S-100 44 Stockholm, Sweden

**Keywords:** strong coupling, nanoantenna, single quantum dot

## Abstract

The strong coupling between single quantum emitters and resonant optical micro/nanocavities is beneficial for understanding light and matter interactions. Here, we propose a plasmonic nanoantenna placed on a metal film to achieve an ultra-high electric field enhancement in the nanogap and an ultra-small optical mode volume. The strong coupling between a single quantum dot (QD) and the designed structure is investigated in detail by both numerical simulations and theoretical calculations. When a single QD is inserted into the nanogap of the silver nanoantenna, the scattering spectra show a remarkably large splitting and anticrossing behavior of the vacuum Rabi splitting, which can be achieved in the scattering spectra by optimizing the nanoantenna thickness. Our work shows another way to enhance the light/matter interaction at a single quantum emitter limit, which can be useful for many nanophotonic and quantum applications.

## 1. Introduction

Recently, the interactions between the emitters and an optical micro/nano cavity have attracted huge interest [1,2,3,4,5]. The strong coupling approaching the single-photon limit between an individual quantum emitter and an optical cavity, which can be described by the cavity quantum electrodynamics (QED), is of particular interest and enables multiple optical applications, such as efficient single-photon sources, quantum information processing [6,7], quantum communication [8,9], low-threshold lasers [10], and ultrafast optical switching [11]. Strong coupling can be manifested in the optical spectrum of the compound structure as a vacuum Rabi splitting. The coupling strength of the interaction is proportional to the ratio of the quality factor of the cavity to the optical mode volume. Therefore, one method to achieve strong coupling is to use dielectric optical cavities of high Q, for example, micropillars [12], photonic crystals [13,14], and metasurfaces [15,16,17]. Realizing such a high Q requires complex experimental fabrication techniques or fastidious experimental conditions. In addition, the coupling strength relies on the local electric field. A higher local electric field leads to greater coupling strength [15]. Cavities made of metal materials, that can support surface plasmons (SPs), can focus the electromagnetic fields into the deep sub-wavelength mode volumes [18,19]. Therefore, utilizing such optical nanocavities can simplify the experimental conditions for strong interactions between light and matter, and will benefit quantum optical experiments carried out under ambient conditions.

At visible wavelengths, noble metals, such as gold and silver, are used to construct micro/nano structures supporting SPs in many applications [20,21,22]. Although the Q factor of an SP mode is relatively low compared with photonic cavities, their optical volume can be drastically reduced. Many research studies have been conducted to study the interactions between SP modes and quantum emitters. In recent years, strong coupling has also been observed by using plasmonic gold dimers [23], silver nanorod [24], and silver nanoprism [25], etc. Hundreds of quantum emitters or more were involved in these experiments. However, for quantum information processing, one needs to reach the limit of a single emitter coupled to the cavity. H. Groß et al. realized the strong coupling between a scanning plasmonic nanoresonator probe and a single semiconductor QD under ambient conditions [26]. K. Santhosh et al. demonstrated that the coupling ratio (of the quality factor of the cavity to the optical mode volume) can be close to the strong coupling regime when a single QD was placed in the nanogap of a silver bowtie [27]. By further increasing the local electric field enhancement and reducing the optical mode volume, one can indeed achieve strong coupling between individual quantum emitters and micro/nanocavities.

In this work, a plasmonic nanoantenna placed on a metal film is proposed, which exhibits ultra-high electric field enhancements in the nanogap and ultra-small optical volumes. Strong coupling between a single quantum dot and the designed structure can be achieved. When a single QD is inserted into the nanogap of the silver nanoantenna, the scattering spectra show a distinct spectral splitting and the vacuum Rabi splitting can be up to 188 meV. Both the finite difference time domain (FDTD) simulations and the coupled-mode theory (CMT) analysis confirm that the strong coupling regime is approached in the hybrid system. This work offers a new way to fulfil strong light-matter interactions at the single quantum emitter limit, which can be useful for quantum and nanophotonic applications.

## 2. Materials and Methods

The scattering spectra of the silver nanoantenna placed on the silver film and the substrate, as well as the hybrid structures, were numerically simulated by using three-dimensional (3D) FDTD (Lumerical FDTD Solutions).

The refractive index of silver was obtained from the data provided by Johnson and Christy [28]. The permittivity of the QDs can be approximated using a Lorentz oscillator model:

(1) $$\varepsilon (\omega )=\varepsilon_{\infty }+\frac{f\omega_{0}^{2}}{\omega_{0}^{2}-\omega^{2}-i\Gamma_{0}\omega }$$

where ε_∞_, f, ω_0_ and Γ_0_ are the high-frequency dielectric constant, the oscillator strength, the exciton transition frequency, and the exciton linewidth, respectively. In this work, these parameters were chosen as ε_∞_ = 6.1, f = 0.6, ω_0_ = 1.879 eV, and Γ_0_ = 80 meV [27].

A total-field scattered-field (TFSF) source with a polarized transverse electric (TE) field (polarization along the y-axis) is used to calculate the scattering spectra of the designed structures. Perfectly matched layer (PML) boundary conditions were employed surrounding the whole structures. A finer mesh region with a size of 0.5 × 0.5 × 0.5 nm^3^ was applied in the nanogap while a coarser mesh with a size of 1 × 1 × 1 nm^3^ was applied elsewhere.

## 3. Results and Discussion

### 3.1. The Proposed Plasmonic Nanoantenna on a Metal Film

**Figure 1:**
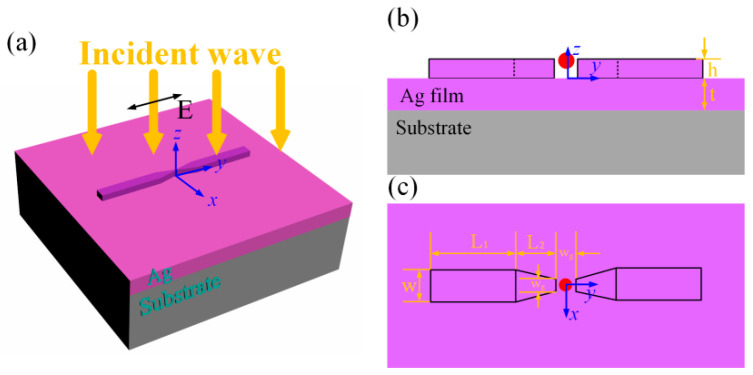
(**a**) Schematic diagram of the proposed silver nanoantenna on a silver film. (**b**) Side view of the structure in the y-z plane. (**c**) Top view of the structure in the x-y plane.

Figure 1 schematically shows the designed structure, which comprises the plasmonic nanoantenna, a metal (silver) film, and a substrate. The perspective, side, and top view of the structure are depicted in Figure 1a–c, respectively. The coordinate system and parameters of the structure are also given in these figures. As shown in Figure 1a, the plasmonic nanoantenna is made of two identical parts, which both have a cuboid and four frustums of a pyramid. The parameter denotations are shown in Figure 1b,c. The thickness of this nanoantenna is h. A nanogap is formed between two parts of the nanoantenna with length g and width w_g_. These two parameters have the same values. The length and width of the rectangle is L_1_ and w, and the height of the trapezoid is L_2_. In this work, the material of the plasmonic nanoantenna is chosen as silver due to its relatively low losses and a higher quality factor Q when compared with other metal materials. The thickness of the silver film is t = 200 nm, which is thick enough to block the incident wave to transmit the structure. The above structure is placed on top of the silica (n = 1.47) substrate. By varying the structural parameters of the nanoantenna, the resonance of the localized SP can be tuned. In order to support the resonant mode with ultra-large electric field enhancements and ultra-small optical volumes in the nanogap, the parameters of the silver nanoantenna were chosen as follows: L_1_ = 370 nm, L_2_ = 126 nm, w = 70 nm, and w_g_ = 7 nm. Consequently, when a single quantum emitter was inserted into the nanogap, strong coupling between the plasmonic nanoantenna and the quantum emitter can be achieved.

**Figure 2:**
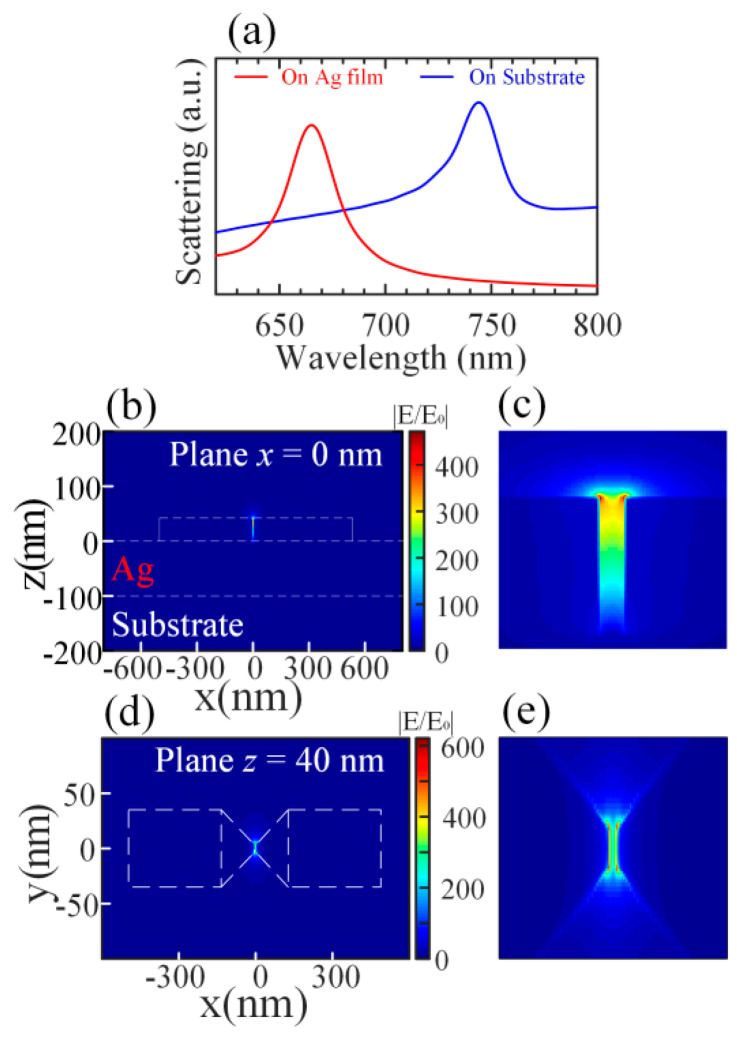
(**a**) Simulated scattering spectra of the silver nanoantenna placed on a silver film (red line) and directly on a silica substrate (blue line). (**b**) The electric field distribution of the longitudinal mode on the plane x = 0 for the silver nanoantenna placed on the silver film. (**c**) The magnified electric field distribution in the nanogap in (**b**). (**d**) The electric field distribution of the longitudinal mode on the plane z = 40 nm for the silver nanoantenna placed on the silver film. (**e**) The magnified electric field distribution in the nanogap in (**d**). The color bars indicate the electric field enhancement.

The plasmonic behavior of the single silver nanoantenna placed directly on a silver film and a substrate was investigated by using the finite-difference time-domain (FDTD) method (Lumerical FDTD Solutions). The detailed simulations can be found in the Materials and Methods section. The calculated scattering spectra are depicted in Figure 2a. From this figure, under both conditions, the structure possesses longitudinal mode due to the dipolar coupling between the two parts of the silver nanoantenna. When placed on the silica substrate, the blue line in Figure 2a shows that the plasmonic nanoantenna supports a localized SP mode at 744 nm and the Q factor is 12.6. However, for the nanoantenna on the silver film, the resonant frequency of the mode has a blue shift and exhibits a higher Q factor, i.e., 22.2. The electric field distributions at the plane x = 0 for the nanoantenna placed on the silver film is shown in Figure 2b. It can be seen clearly that the electric field is highly confined in the nanogap of the nanoantenna. Figure 2c shows the enlarged electric field distributions in the nanogap. Furthermore, the electric hot spot is concentrated at the top of the nanogap. The electric field is maximized at the vicinity of the nanoantenna, which is enhanced as high as 470-fold. There is no noticeable electric field enhancement in the other parts of the structure. The electric field distributions at the top plane (z = 40 nm) of the nanoantenna are drawn in Figure 2d. Similarly, the enlarged electric field distributions in the nanogap at this plane are shown in Figure 2e. When getting close to the four vertexes of the nanogap, the electric field is greatly enhanced and can be more than 600 times the incident wave. As depicted in Figure 2e, the electric field will be enhanced more than 200 times in the center of the nanogap. The calculated optical volume of the resonant longitudinal mode is ultra-small, only 1.61 × 10^−6^ λ^3^ (due to the highly confined electric field in the nanogap), which is one order lower than that of a plasmonic waveguide–slit structure on a metallic substrate reported by G. Zhang et al. [29].

**Figure 3:**
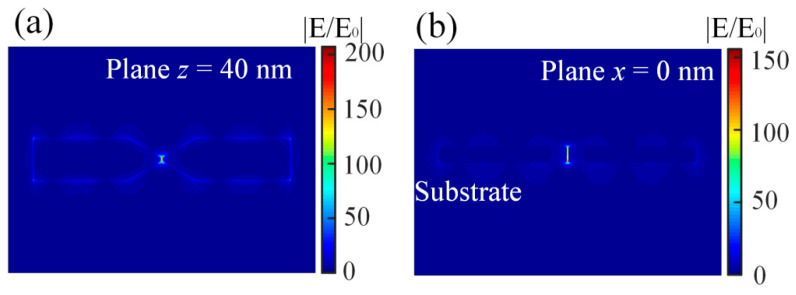
(**a**) The electric field distribution of the longitudinal mode on the plane z = 0 for the silver nanoantenna directly placed on the silica substrate. (**b**) The electric field distribution of the longitudinal mode on the plane x = 0 for the silver nanoantenna directly placed on the silica substrate. The color bars indicate the electric field enhancement.

In addition, the electric field distributions at the plane x = 0 of the nanoantenna placed on the silica substrate are shown in Figure 3a. The electric field is highly focused in the nanogap as well. However, when compared with Figure 2b, the electric hot spot is concentrated at the bottom of the nanogap, which is different from the nanoantenna placed on the silver film. The electric field distributions at the bottom plane (z = 0 nm) of the nanoantenna are depicted in Figure 3b. The electric field in the nanogap is the strongest and the electric field enhancements are observed surrounding the nanoantenna and the silica substrate. In the plane x = 0 nm, the electric field is enhanced at a maximum more than 150 times and this is much lower than that of the silver nanoantenna placed on the silver film. It can be found that the silver film can help the concentrations of the electric fields in the nanogap of the nanoantenna.

**Figure 4:**
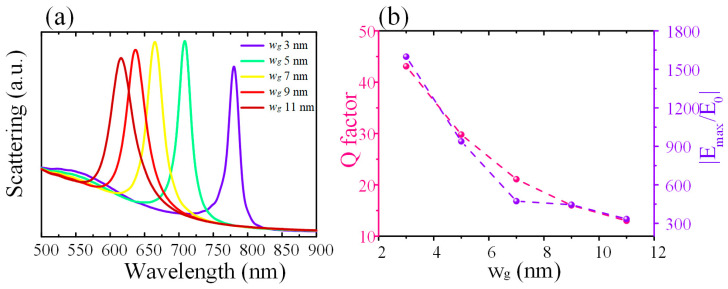
(**a**) The simulated scattering spectra of the nanoantenna on the silver film with different values of the nanogap. (**b**) The corresponding Q factor and the maximum electric field enhancements of the scattering spectra with different values of the nanogap.

Local electric field enhancement is important to realize the strong coupling between light and matter. The effects of the size of the nanogap on the Q factor and the local electric field enhancements were studied numerically and the results are shown in Figure 4. Figure 4a is the relation between the calculated scattering spectra on the excitation wavelength and the nanogap size w_g_ (from 3 nm to 11 nm). When w_g_ increases from 3 nm to 11 nm, the linewidth of the resonance increases and the resonance exhibits a blueshift. This means that the corresponding Q factor decreases when the size of the nanogap w_g_ increases. The obtained Q factors and the maximum electric field enhancement (|E_max_/E_0_|) for different nanogap sizes w_g_ are depicted in Figure 4b. When w_g_ is 3 nm, the Q factor of the resonance is 43.1 and the maximum electric field enhancement can be as high as 1599. The Q factor and the maximum electric field enhancement then decrease with the increase of w_g_. For example, when w_g_ is 11 nm, the Q factor only reaches 13.9 and the maximum electric field enhancement is 334. Therefore, one can vary the size of the nanogap to study the light and matter interactions in the nanogap of the silver nanoantenna. The proposed nanoantenna can be fabricated by electron beam lithography (EBL), metal deposition, and liftoff, arranged in an appropriate sequence. The gap can be as small as 8 nm by using the advanced EBL process [30]. Atomic layer lithography was developed to create patterned nanogaps in metallic structures via atomic layer deposition (ALD) [31]. Because the lateral dimension is defined by ALD in this method, the fabricated gaps can be as small as 1 nm.

### 3.2. Strong Coupling between the Antenna and Single QD

**Figure 5:**
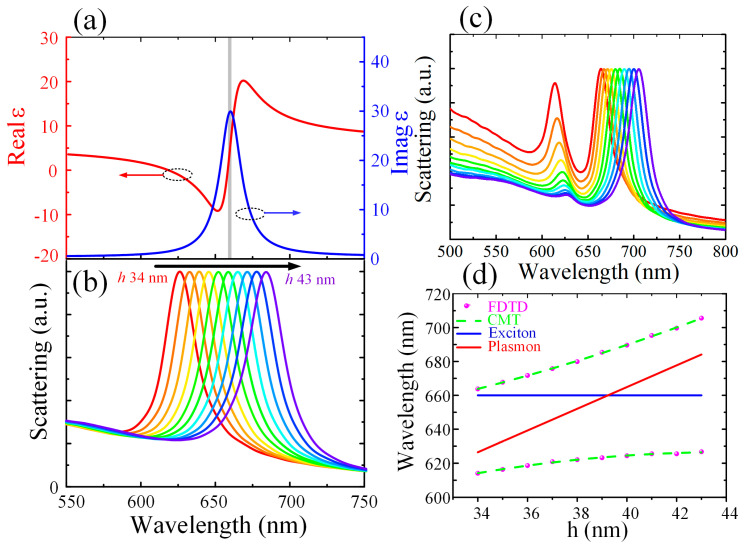
(**a**) The real (red line) and imaginary (blue line) parts of the relative permittivity of the single QD used in this work. (**b**) Simulated scattering spectra for different thicknesses h of the silver nanoantenna placed on the silver film. (**c**) Simulated scattering spectra for different thicknesses h of the silver nanoantenna coupled with the single QD in the nanogap. (**d**) The resonance of the two new coupling states as the silver nanoantenna thickness h varies. The purple dots and green dashed curve correspond to the FDTD simulation and the CMT results, respectively. The blue and red curves stand for the individual resonances of the exciton and the plasmon modes, respectively.

In this work, the QD with an exciton transition at 660 nm was modeled by a Lorentz oscillator model, which was detailed in the Materials and Methods section. The calculated permittivity of the QD is displayed in Figure 5a. A series of silver nanoantennas was designed with the thickness h ranging from 34 nm to 43 nm in 1 nm steps, to tune the bare plasmon resonance from approximately 627 nm to 684 nm, covering the exciton resonance of the QD. Figure 5b shows the scattering spectra of the silver nanoantenna with different thicknesses. It can be found that the scattering spectra show a redshift while the Q factor remains unchanged when the thickness increases from 34 nm to 43 nm.

As shown in Figure 1, a single QD with a diameter of 6 nm was inserted into the top center of the nanogap, which then led to great electric field enhancements. Two methods can be utilized to insert a single QD into the nanocavity. One method is to use interfacial capillary forces to drive the QDs even to the single limit into the nanogaps, as presented by Santhosh et al. [27]. The other method takes the advantage of atomic force microscopy (AFM) to manipulate a single QD into the nanocavity [32,33]. Figure 5c shows the scattering spectra of the mixed structure with different silver nanoantenna thicknesses h. As the curves in Figure 5b show, the resonance of the plasmon increases when the thickness h increases. As the thickness h increases, the resonance of the plasmon mode will shift across the exciton resonance. As shown in Figure 5c, all the scattering spectra show a clear mode splitting with two new peaks, which are different from the individual plasmon and exciton resonances. The scattering peaks are the result of the strong light-matter interactions between the plasmon and the exciton resonances.

The purple dots and green dashed curve correspond to the FDTD simulation and the CMT results, respectively. The blue and red curves represent the individual resonances of the exciton and the plasmon modes, respectively. As shown in the scattering spectra in Figure 5d, unique anticrossing upper (UB) and lower (LB) bands are obtained. Such an anticrossing trend proves the robustness of the nanogap-exciton coupling in the hybrid structure. Coupled mode theory (CMT) was used to describe the two new states and was fitted for the exciton-plasmon coupling dispersion. The eigen energies of the coupling modes E_LB,UB_ are given by [34]:

(2) $$\left[\begin{array}{cc} E_{p}+i\hbar \gamma_{p} & g\\ g & E_{e}+i\hbar \gamma_{e} \end{array}\right]\left[\begin{array}{c} \alpha \\ \beta \end{array}\right]=E_{LB,UB}\left[\begin{array}{c} \alpha \\ \beta \end{array}\right]$$

Here, E_p_ and E_e_ are the energies for the bare plasmon mode and the exciton resonance, respectively. γ_p_ and γ_e_ are the half-bandwidths of the bare plasmon and the exciton resonances, respectively. g is the coupling rate characterizing the interaction between the plasmon mode and the exciton resonance. α and β are the Hopfield coefficients, which meet |α|^2^ + |β|^2^ = 1. |α|^2^ and |β|^2^ stand for the fractions of the plasmon mode and the excitons in the new states, respectively. The eigenvalues are achieved as:

(3) $$E_{LB,UB}=\frac{1}{2}[E_{e}+E_{p}+i(\gamma_{e}+\gamma_{p})/2]\pm \sqrt{g^{2}+\frac{1}{4}{\left[E_{e}-E_{p}+i(\gamma_{p}-\gamma_{e})\right]}^{2}}$$

Using Equation (3), with zero detuning for the two new hybrid bands, the Rabi splitting energy of:

(4) $$\hbar \Omega =2\sqrt{g^{2}-{\left(\gamma_{p}-\gamma_{e}\right)}^{2}/4}$$

can be obtained from the FDTD and the CMT fitting results in Figure 5d. To meet the strong coupling criteria, one of the following conditions should be satisfied:

(5) $$N_{1}=\hbar \Omega /(\gamma_{p}+\gamma_{e})>1\;\mathrm{and}\;N_{2}=g/\sqrt{(\gamma_{e}^{2}+\gamma_{p}^{2})/2}>1$$

where γ_e_ = 40 meV and γ_p_ = 42.0 meV. The Rabi splitting ħΩ = 188.0 meV at zero detuning is extracted from the FDTD calculated results. An interaction strength g = 100.8 meV was obtained. Thus, from the FDTD simulations, N_1_ = 2.29 and N_2_ = 2.46 were calculated, which satisfy the strong coupling criteria. According to the CMT, the dispersion of the exciton-polaritons can be fitted and the results are plotted as shown in Figure 5d. From the fitting curve, we can obtain g = 99.5 meV and ħΩ = 185.1 meV, hence N_1_ = 2.20 and N_2_ = 2.43, which will satisfy the strong coupling criteria as well. Therefore, both the CMT and the FDTD results confirm that strong coupling is achieved. In addition, the Rabi splitting can be further increased when the QD is inserted very closely to the silver nanoantenna.

## 4. Conclusions

In summary, a plasmonic nanoantenna placed on a metal film, which has an ultra-high electric field enhancement and an ultra-small optical volume in the nanogap, was designed. When compared with the nanoantenna directly placed on the substrate, the metal film contributes to increase the local field enhancement further. The strong coupling between the silver nanoantenna placed on a metal film and a QD has been confirmed both theoretically and numerically. The simulation results show strong coupling between the exciton and the plasmon modes in scattering spectral splitting. Furthermore, the anticrossing behavior with the two new states can be achieved by changing the thickness of the silver nanoantenna. The unique coupling can also be used to realize strong light−matter interactions in a single quantum emitter, and the hybrid structure designed here will be beneficial to quantum information operations and other nanophotonic applications.

## Data Availability

Not applicable.
